# Oculomotricity parameters in digital nystagmography among children with and without learning disorders

**DOI:** 10.1016/S1808-8694(15)30526-7

**Published:** 2015-10-18

**Authors:** Denise de Fátima Pires Ventura, Lídio Ganato, Edson Ibrahim Mitre, Rita Mor

**Affiliations:** 1 MSc in Health Sciences - Santa Casa de São Paulo, Prof. of Clinical Audiology - CEFAC- Saúde e Educação; 2 PhD in Medicine. Adjunct Professor - Department of Otolaryngology - Medical Sciences College -Santa Casa - São Paulo; 3 PhD in Medicine, Attending Physician - Otolaryngology Department - Medical Sciences College -Santa Casa - São Paulo; 4 MSc in Human Communication Disorders – UNIFESP, Coordinator of the Audiology and Neurotology Department - CEFAC- Saúde e Educação. Santa Casa de São Paulo/ CEFAC- Saúde e Educação

**Keywords:** learning, labyrinth diseases, vertigo

## Abstract

The saccadic pathway involves numerous regions of the brain cortex, the cerebellum and the brainstem. Saccadic movement latency, velocity and precision parameters assess the efficacy of central nervous system (CNS) control over rapid eye movements. Very few disorders which alter the CNS are missed when these parameters are carefully measured using a computer. Pendular tracking assesses the integrity of the oculomotor system in controlling slow eye movements - vulnerable to CNS and vestibular system dysfunctions. Optokinetic nystagmus represents a stereoceptive response which compensates environment movements by psycho-optical inputs.

**Aims:**

to compare the oculomotricity values found in children with and without learning complaints.

**Materials and Methods:**

prospective study. We included in the study 28 children of both genders, within the age range between 8 and 12 years, with learning disorders (study group) and 15 without (control group). We carried out the fixed and randomized saccadic movement tests, pendular tracking study and optokinetic nystagmus.

**Results:**

There was a statistically significant difference between the groups concerning the randomized saccadic movement velocity parameters and in the pendular tracking test.

**Conclusion:**

The children with learning disorders presented alterations in some oculomotricity tests when compared to children without complaints.

## INTRODUCTION

Saccade voluntary control abnormalities have been seen in development disorders such as: dyslexia; learning disorders; hyperactivity and attention deficit. The saccadic pathway involves numerous regions of the brain cortex, cerebellum and brainstem. Latency, velocity and saccadic movement precision parameters assess the central nervous system (CNS) control efficiency over rapid eye movements^1^. There are just a handful of disorders which affect the CNS and can not be detected even when these parameters are measured precisely by a computer. Mild alterations in fixed and randomized saccadic movement latency, velocity and precision parameters do not necessarily indicate a central disorder. Pendular tracking is another eye movement resulting from the tracking of a mobile target and assesses the oculomotor system in the control of slow eye movements, and it is vulnerable to CNS and vestibular system dysfunctions. The optokinetic nystagmus is a rhythmic, involuntary, unconscious and automatic ocular phenomenon. It can be reproduced by following points which move in one direction and afterwards in the opposite direction. It represents an exteroceptive response which makes up for environmental movements by psycho-optic impulses. Pendular tracking and the optokinetic nystagmus use the same final nervous pathway, stemming from different origins. Some authors advocate that when the pendular tracking is normal, the optokinetic test can be ommited.^2^, ^3^, ^4^

Pendular tracking is a test which is strongly affected by the patient's attention span and collaboration. There may be cases of pendular tracking poorly formed in inattentive and non-cooperating patients or even in some elderly individuals, without it meaning a central disorder^3^.

The necessary eye movement for reading requires alternate saccade movements and fixation periods. It starts with a saccade that runs for 8 to 10 words mixed with periods of eye fixation and ends with a long saccade in order to start a new line^4^, ^5^, ^6^, ^7^, ^8^, ^9^. They are used in order to follow the teacher within the visual field, in the classroom, to make copies, transcribe lessons written down on the board, read textbooks, to write and concentrate on activities which require integrity of the oculomotor functions and vestibular interconnections^7^.

The goal of the present study was to compare the eye movement parameters found in children with learning disorders - especially in reading and writing - with children without complaints.

## MATERIALS AND METHODS

**QUESTIONNAIRE TEMPLATE**

Child: ________________________________________________________________

Current date: ____/____/ ________

Parent/guardian: ________________________________________________________

The questionnaire below aims at studying learning difficulties. Please, answer it by checking with a × in the option you think correct and fill in the blanks when requested. The data hereby expressed serve research purposes (data computing) and will be kept confidential. It is very important that you return this form to us. The questionnaire must be returned on the day of the medical consultation.

Does your child have or has had:

Difficulties reading: yes ( ) no ( ) - Which? _____________________________

Difficulties writing: yes ( ) no ( ) - Which? _____________________________

Poor school performance: yes ( ) no ( ) Repeated a school year (s): yes ( ) no ( )

Why do you think this happened? ______________________________________________

_____________________________________________________________________________

Problems of attention and concentration at school: yes ( ) no ( )

The population studied was made up of 43 children of both genders, of age range between 8 and 12 years, being 28 (study group) with diagnosis of learning disorders (reading and writing) and 15 belonging to the control group without any learning disorder. All the children, from both groups, did not wear glasses. We carried out fixed and randomized saccadic movement test, pendular tracking test in the frequencies of 0.20Hz, 0.40Hz and 0.80Hz and optokinetic nystagmus test. Equipment: digital vectonystagmophapher with the VECWIN software, a Neurograff Eletromedicina® bar of led. The parents/guardians received a questionnaire about learning-related symptoms. The analyses were automatically done by the software. As far as eye movements are concerned, we analyzed the following parameters: accuracy (it is the precision with which the patient's eyes track the movement of a point of light on the visual stimulation bar, controlled by the equipment); velocity (assess saccade intensity, expressed in degrees per second); latency (time interval in milliseconds between the target movement and the eyes reaction, saccade, of the patient) and gain (measure the relationship between eye velocity and the stimulus velocity used in optokinetic and pendular tracking tests)^10^. In order to characterize the sample we carried out a descriptive analysis. For the quantitative variables we present some summary measures and for the qualitative variables we present tables with frequency and percentage. In order to compare the quantitative variables among the group we employed the ANOVA variance analysis, for the qualitative analysis the Fisher Exact Test and the t-paired test to compare the sides. The level of significance (p) used was 5%. For the statistical analysis e used the EPI-INFO® version 3-3.2. and SPSS® for Windows version 13.0. This study was developed at the Clinical Audiology Sector of the Clinical Speech and Hearing Specialization Center - Centro de Especialização em Fonoaudiologia Clínica - CEFAC Saúde e Educação, São Paulo, after being approved by the Ethics in Research Committee of this institution, on June 13, 2005, under protocol # 081/05. Both children and guardians were informed about the procedures. The assessment protocol was only employed after the subjects' guardians agreed to their participation in the study and signed a free and informed consent form.

## RESULTS

**Figure 1:**
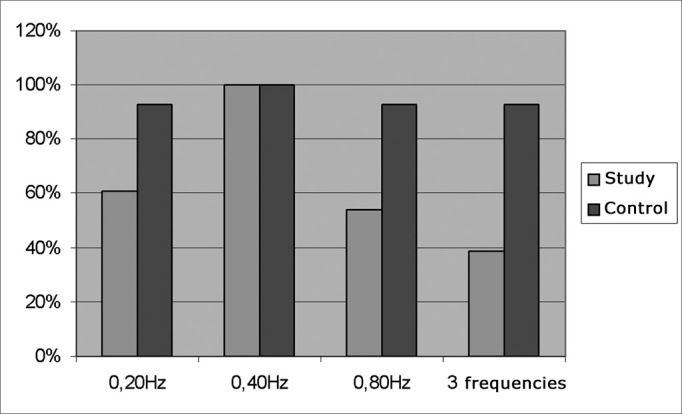
Pendular tracking - Percentage of children who were able to perform the pendular tracking test in the frequencies of 0.20Hz; 0.40Hz; 0.80Hz in the three frequencies. Relationship between the groups.

As far as gender is concerned the control and study groups were homogeneous (p= 0.518). The distribution is on Table 1. Table 2 shows some age summary measures. Group comparison did not show statistically significant differences (p= 0.141). Of the 28 children in the study, 24 (86%) presented reading difficulties symptoms, 21 (75%) had poor school performance, 16 (57%) had lack of attention and concentration, 6 (21%) repeated school years. None of the control group children had any of the aforementioned symptoms. In terms of the eye movement tests, there was a statistically significant difference between the groups concerning the velocity of randomized saccade movement parameters when looking to the right (p= 0.035) (Table 3). In the pendular tracking study of the control group, 93% of the children were able to perform the tests in the three frequencies presented; and in the study group 39% of the children did the test (Fig. 1). There was a statistically significant difference between the groups in terms of being able to perform the test in the frequency of 0.20Hz (p equals; 0.023) and in the frequency of 0.80Hz (p equals; 0.008). The gain measures in pendular tracking in the frequencies assessed did not show statistically significant differences in comparing the groups (Table 4). As to the optokinetic nystagmus, it was present in both directions in the light bar visual stimulation in all the children of the control group (100%). In the study group there were 27 children (96%) and 1 child (4%) was not able to do it. There was no statistically significant difference between the slow component angular velocity (SCAV), optokinetic nystagmus symmetry and gain among the groups (Table 5, Table 6, Table 7).

**Table 1:** Gender distribution of the control and study groups.

	control	%	study	%	TOTAL	%
Males	9	60	18	64	27	63
Females	6	40	10	36	16	37
TOTAL	15	100	28	100	43	100

**Table 2:** Summary values of the group ages.

Group	n	mean	Standard deviation	minimum	maximum	median
Control	15	9,4	1	8	12	9
Study	28	8,9	1	8	11	9

**Table 3:** Comparing the randomized saccade movements between the groups.

Velocity	Group	n	Mean	Standard deviation	Descriptive level (p)
Right	Study	28	79,5	13	0,035*
	Control	15	70,3	12	
Left	Study	28	78,7	23	0,483
	Control	15	74	16	

p ≤ 0,05

**Table 4:** Comparing the study and control groups in pendular tracking gain in the frequencies of 0.20; 0.40 and 0.80Hz.

Frequency	Group	n	Mean	Standard deviation	Descriptive level (p)
0,20Hz	Study	17	0,9	0,2	
	Control	14	0,9	0,2	0,930
0,40Hz	Study	28	1,0	0,2	
	Control	15	1,0	0,2	0,612
0,80Hz	Study	15	0,9	0,2	
	Control	14	0,9	0,2	

p ≤ 0,05

**Table 5:** Comparing the optokinetic nystagmus slow component angular velocity between the groups:

SCAV	Grupo	n	Mean	Standard deviation	Descriptive level (p)
Right	Study	27	9,7	2,7	0,773
	Control	15	9,9	2,0	
Left	Study	27	10	2,2	0,626
	Control	15	9,8	1,6	

p ≤ 0,05

**Table 6:** Comparing the optokinetic symmetry between the groups

PDN	Group	n	Mean	Standard deviation	Descriptive level (p)
Symmetry	Study	27	5,8	3,9	0,232
	Control	15	4,3	3,8	

p≤0,05

**Table 7:** Comparing the optokinetic nystagmus gain to the right and to the left in the study and control groups

Group	n	Mean difference	Standard deviation	Descriptive level (p)
Study	28	0.06	0.15	0.059
Control	15	0.05	0.1	0.062

p ≤ 0,05

## DISCUSSION

In our study we could notice that the mean values found in fixed saccade movements are within normal ranges in digital vectonystagmography in terms of accuracy, latency and velocity^10^. The difference we found between the groups in random saccadic movement velocity parameters may suggest a possible control failure in the central nervous system in regards of the rapid eye movements^1^, ^2^, ^3^, ^4^. We noticed that in the control group, 93% of the children were able to perform the test in the three frequencies presented and in the study group, 39% of the children did it. The difference between the groups in being able to perform the test in the frequencies of 0.20Hz and 0.80Hz may be caused by the incomplete development of the pathways which control slow tracking eye movements, and also for being a test that is strongly affected by the person's attention span and collaboration, and there may be cases of poorly formed tracking in inattentive patients and in those who do not cooperate, without it necessarily meaning a central disorder^3^, ^4^. We stress that in our study, 57% of the children were not attentive - which corroborates the findings of other studies regarding pendular tracking. In the optokinetic nystagmus, one child was unable to perform the test, despite numerous attempts - because of tearing, which interfered in the test tracing and because sometimes this child moved the head. In this test we did not observe statistically significant differences in relation to the SCAV, symmetry and gain. Nonetheless, the descriptive level (p) obtained in the optokinetic nystagmus gain is near the significance level, and this fact justifies more studies which could prove the sensitivity of oculomotor tests with digital nystagmography in children with learning disorders. Saccade alterations are common in the peripheral or central vestibular system, suggesting an interaction between visual phenomena and the mechanisms involved in maintaining body balance. As to the prevalence of eye movement alterations, we and other researchers^1^, ^2^, ^4^ found in the digital nystagmography in children with learning disorders, it justifies the systematic inclusion of the assessment of these eye movements during speech and hearing evaluation. Based on what was seen in the present investigation, alterations in some of the eye movement tests can be found in children with learning disorders. Therefore, it is necessary to be careful when considering these eye movement alterations as indicators of brainstem dysfunctions.

## CONCLUSION

Children with learning disorders have alterations in some eye movement tests when compared to “normal” healthy children.
